# Supplementary material for: Graphitic mesoporous carbon-silica composites from low-value sugarcane by-products for the removal of toxic dyes from wastewaters
Source: R Soc Open Sci. 2020 Sep 9;7(9):200438. doi: 10.1098/rsos.200438 (PMC7540760; doi:10.1098/rsos.200438)
Supplement: Raman, XPS, SEM and TGA data for CSC materials [file rsos200438supp1.docx]

Supporting information for:

Graphitic mesoporous carbon-silica composites from low value sugarcane by-products for the removal of toxic dyes from wastewaters

Intuorn Janekarn,^a^ Andrew J. Hunt,^a^ Yuvarat Ngernyen,^b^ Sujittra Youngme,^b^ and Nontipa Supanchaiyamat*^,a^

^a^ Materials Chemistry Research Center, Department of Chemistry and Center of Excellence for Innovation in Chemistry, Faculty of Science, Khon Kaen University, Khon Kaen, 40002, Thailand

^b^ Department of Chemical Engineering, Khon Kaen University, Khon Kaen, 40002, Thailand

* Corresponding author. Tel: +66 43 009700 ext. 12243, Fax: +66 43 202373, E-mail: nontsu@kku.ac.th

**Optimization of molasses to silica ratio**

Different ratios of molasses and silica were investigated in order to reveal the optimum ratio that yield the highest incorporated molasses onto the silica. TGA thermograms of molasses and carbon silica composites conducted under air atmosphere are shown in Figure S1 and Table S1 summarised the analysis results. CSCs demonstrated a two stage degradation process. The first weight loss was related to degradation via dehydration, which occurred below 200°C and the second weight loss related to degradation of carbon leading to the release of gaseous volatiles. The CSC40-10 exhibited the highest loading of molasses onto silica with a %TG decomposition of 35.54% at 453 °C.


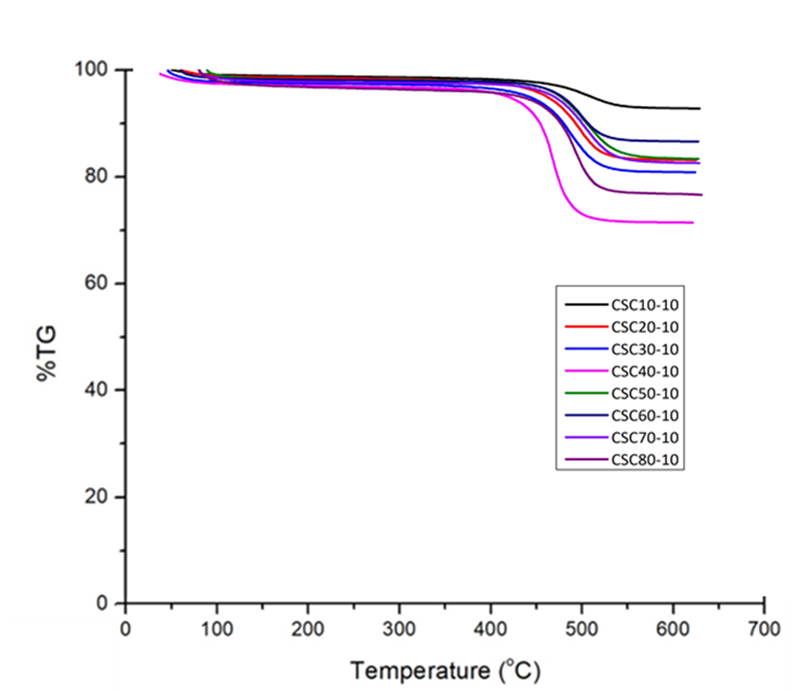


**Figure S1.** TGA thermograms of CSCs at different molasses to silica ratios

**Table S1.** Thermogavomatric analysis results of CSCs at different molasses to silica ratios.

| **Sample** | **Si-60 (g)** | **Molasses (g)** | **%TG** | **Temperature (°C)** |
| --- | --- | --- | --- | --- |
| CSC 10-10 | 10 | 10 | 8.98 | 458 |
| CSC 20-10 | 10 | 20 | 21.03 | 460 |
| CSC 30-10 | 10 | 30 | 23.85 | 451 |
| CSC 40-10 | 10 | 40 | 35.54 | 453 |
| CSC 50-10 | 10 | 50 | 20.79 | 460 |
| CSC 60-10 | 10 | 60 | 16.74 | 457 |
| CSC 70-10 | 10 | 70 | 21.49 | 460 |
| CSC 80-10 | 10 | 80 | 28.99 | 450 |

Moreover, the adsorption-desorption isotherm study indicated that when molasses to silica ratio of 40:10 was used, the lowest pore volume and pore diameter was exhibited, suggesting the highest incorporated molasses onto the silica.

**Table S2.** Textural properties of silica gel 60 and CSC materials prepared from different Si-60/molasses ratio characterized by N_2_ adsorption-desorption isotherm.

| **Sample** | **BET Surface area (m^2^/g)** | **Pore volume (m^3^/g)** | **Pore diameter (Å)** |
| --- | --- | --- | --- |
| Si-60 | 308 | 0.739 | 96.01 |
| CSC 10-10 | 278 | 0.637 | 91.67 |
| CSC 20-10 | 241 | 0.404 | 66.97 |
| CSC 30-10 | 235 | 0.382 | 65.10 |
| CSC 40-10 | 217 | 0.219 | 40.34 |
| CSC 50-10 | 203 | 0.382 | 75.12 |
| CSC 60-10 | 208 | 0.334 | 63.85 |
| CSC 70-10 | 230 | 0.338 | 58.75 |
| CSC 80-10 | 227 | 0.333 | 58.73 |


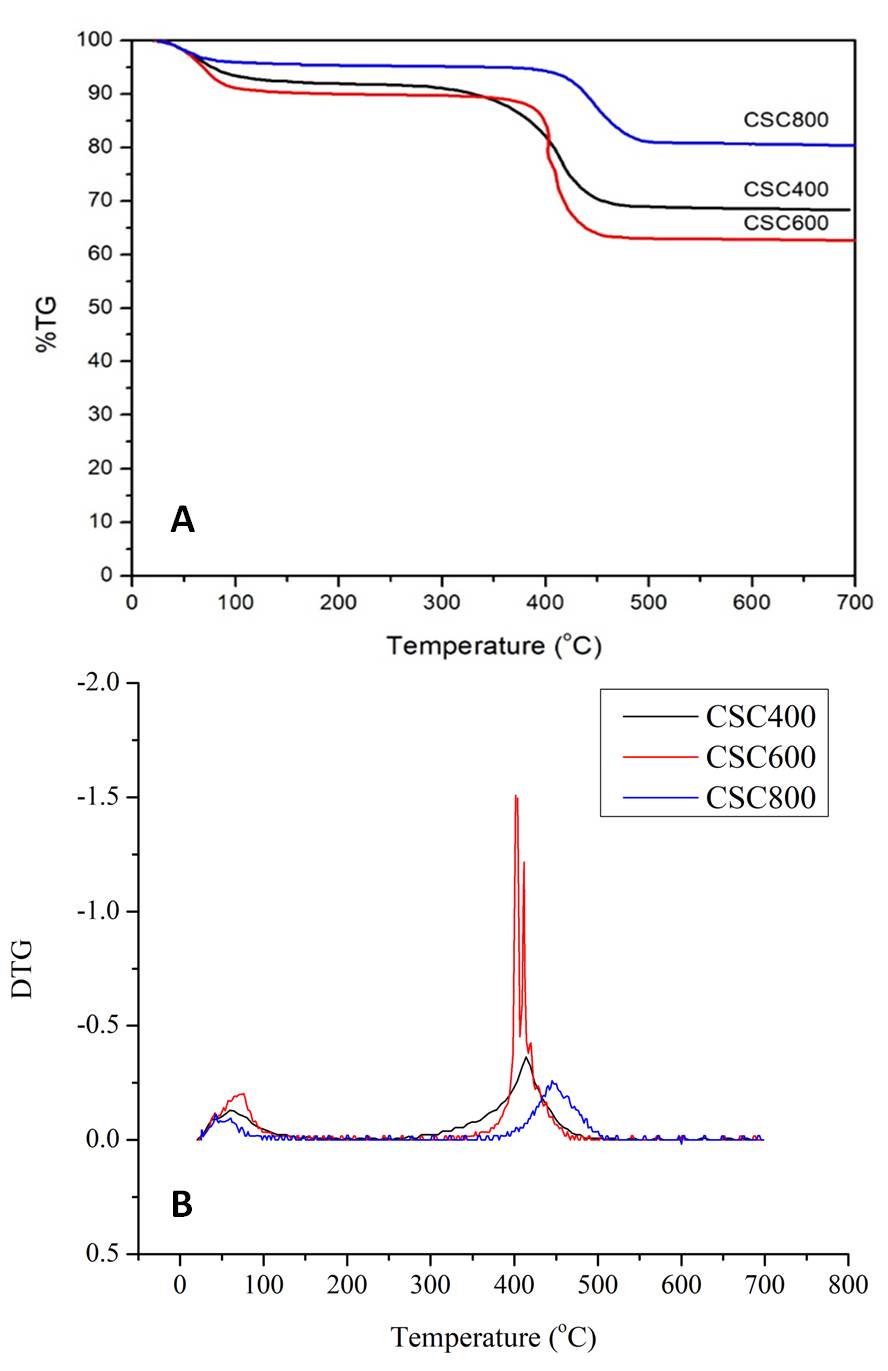


**Figure S2.** TGA (A) and DTA (B) of CSC materials in air.

**Table S3.** TGA parameters of CSCs in air.

| **Parameter** | **CSC400 (^o^C)** | **CSC600 (^o^C)** | **CSC800 (^o^C)** |
| --- | --- | --- | --- |
| T_onset_ | 298 | 362 | 393 |
| T_d_ | 423 | 415,435 | 448 |
| %mass loss (300-500 °C) | 24 | 28 | 14 |
| %char | 68 | 62 | 81 |


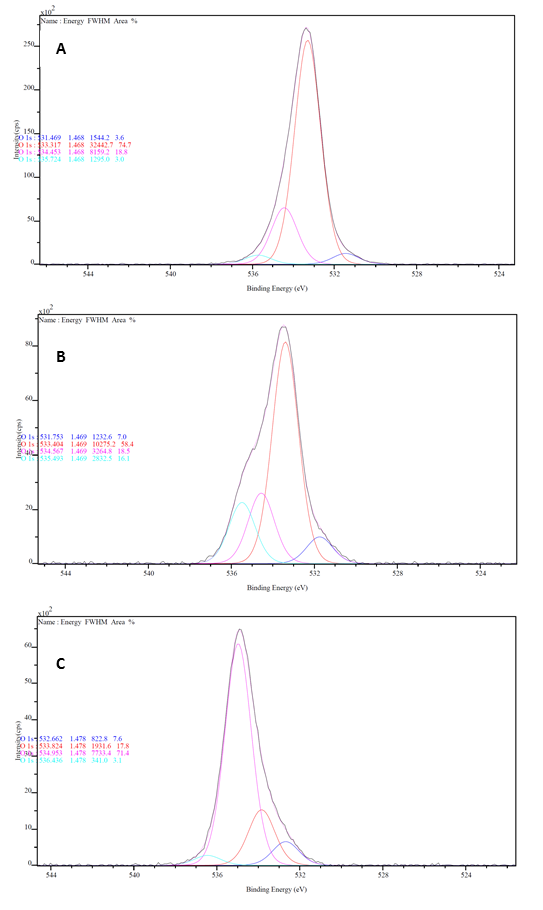


**Figure S3.** O1s XPS spectra of A) CSC400, B) CSC600 and C) CSC800.


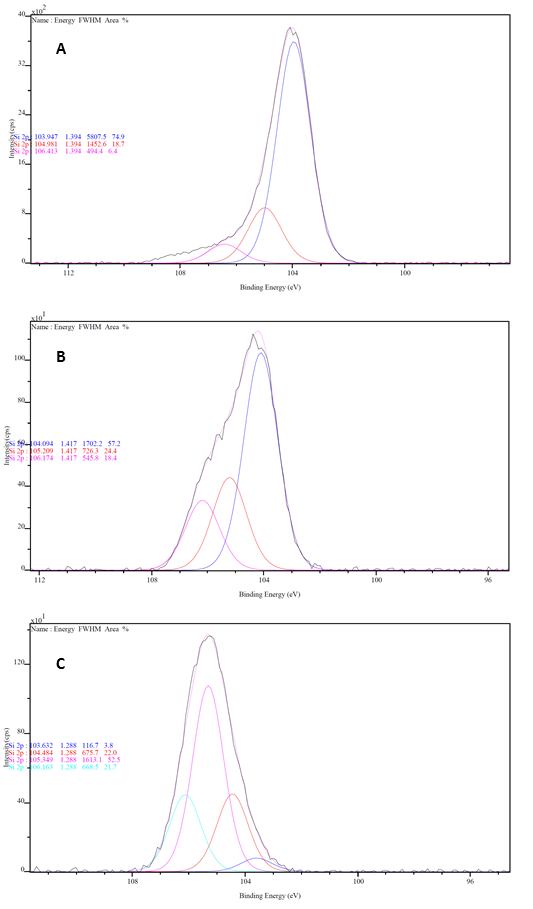


**Figure S4.** Si2P XPS spectra of A) CSC400, B) CSC600 and C) CSC800.


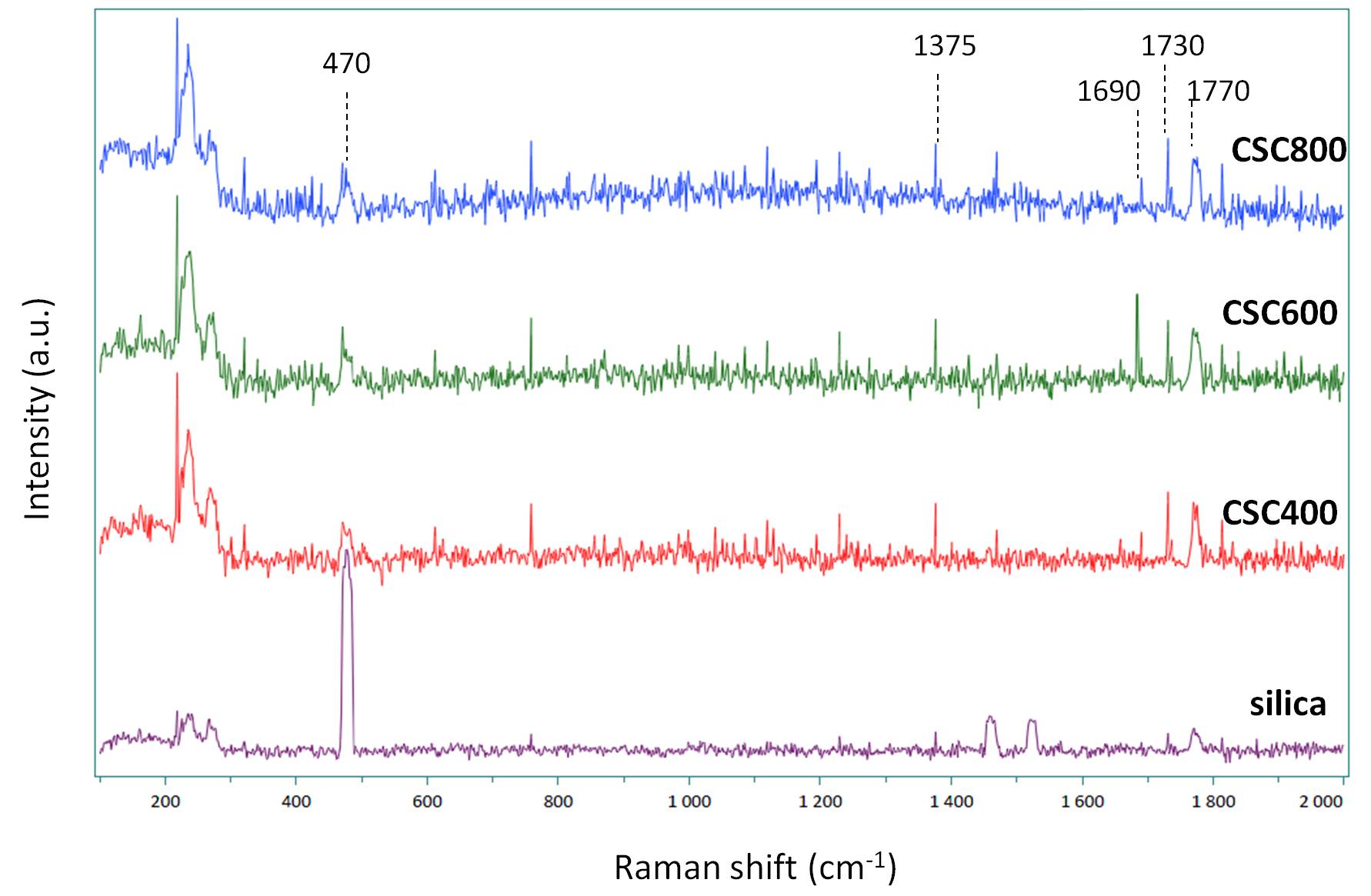


**Figure S5.** Raman spectra of CSCs and silica.


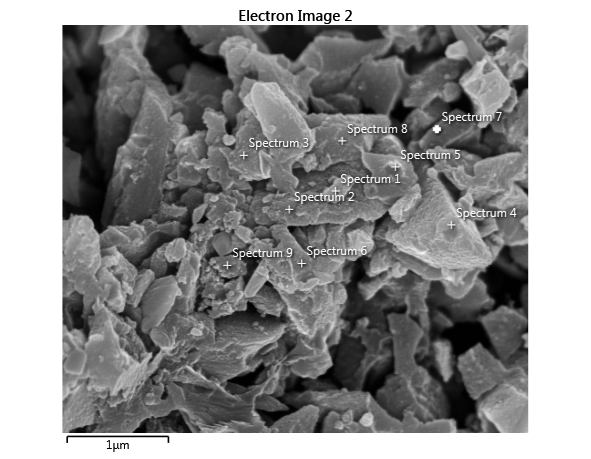


**Figure S6.** SEM-EDS mapping for CSC400.


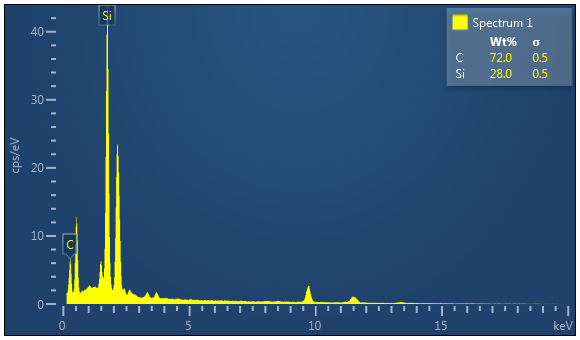

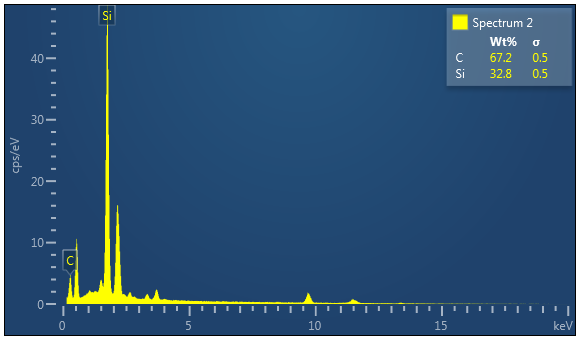

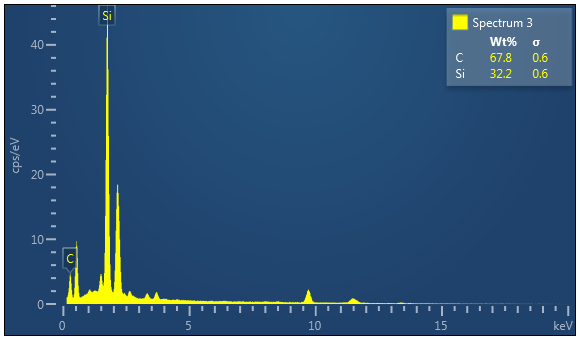

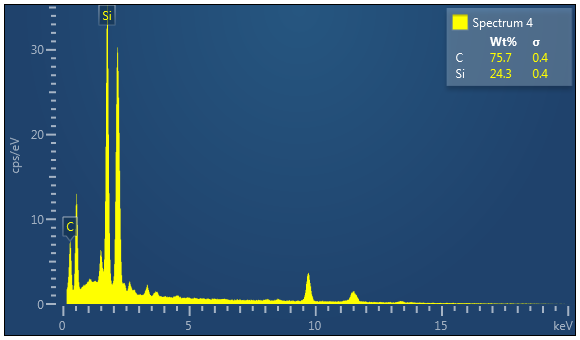

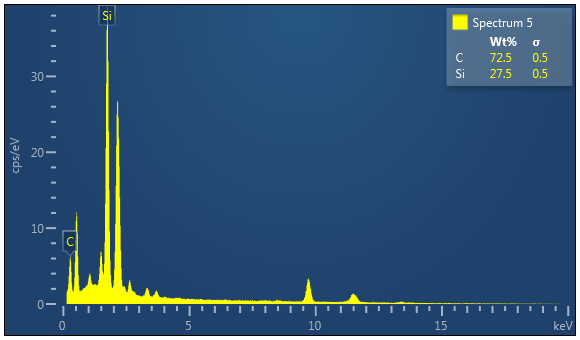

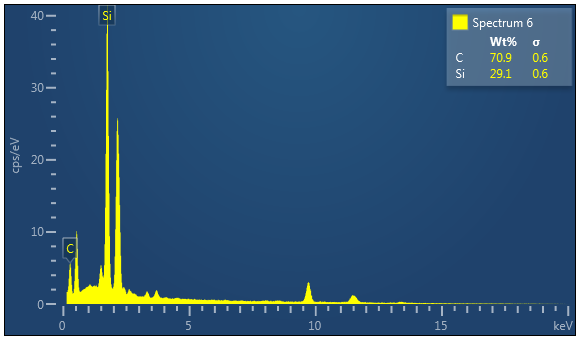

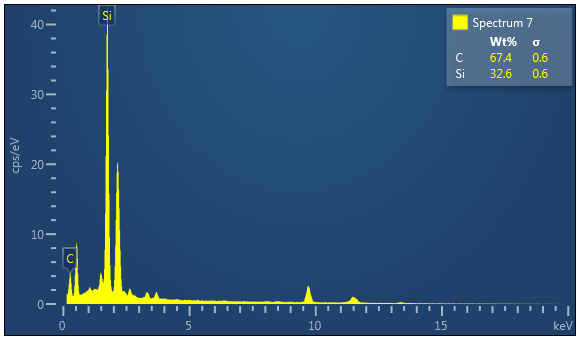

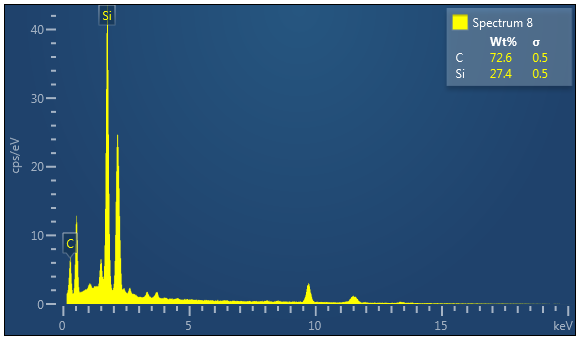

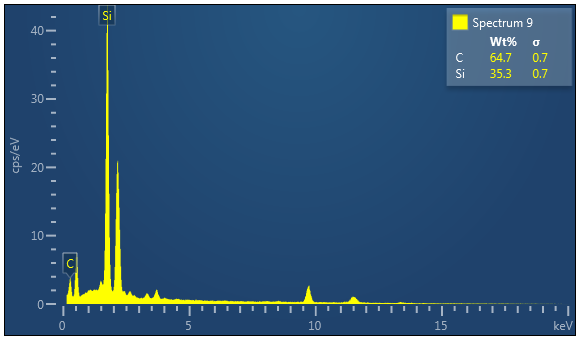


**Figure S7.** Elemental composition as determined by SEM-EDS mapping for CSC400.


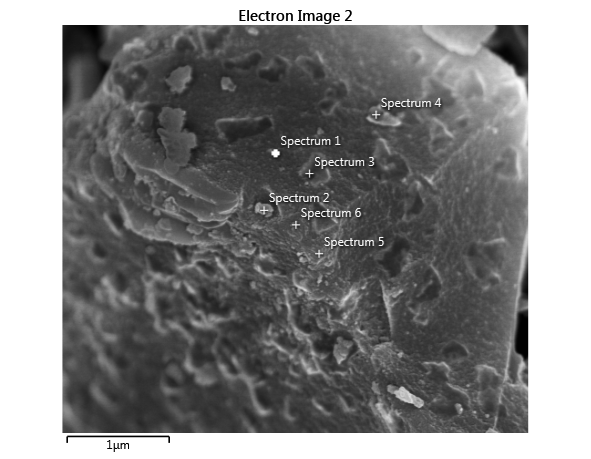


**Figure S8.** SEM-EDS mapping for CSC600.


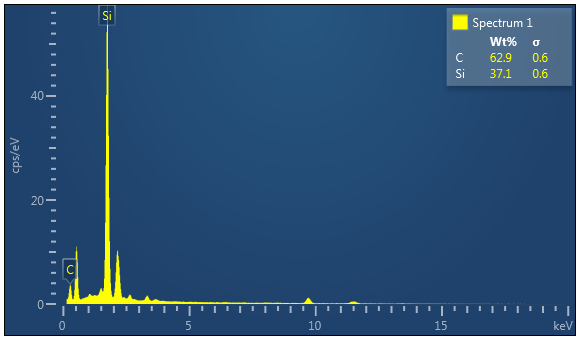

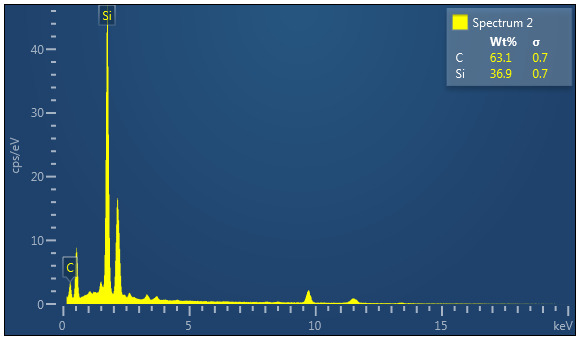

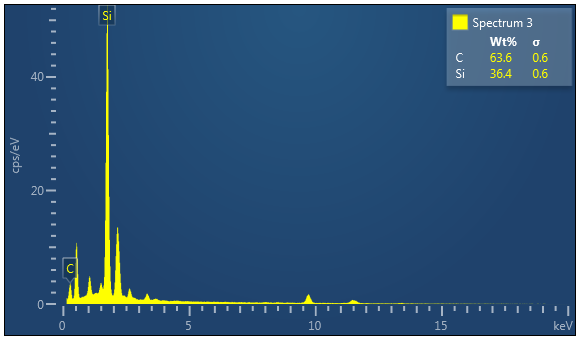

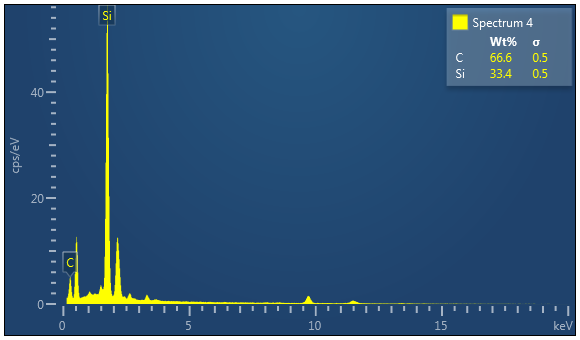

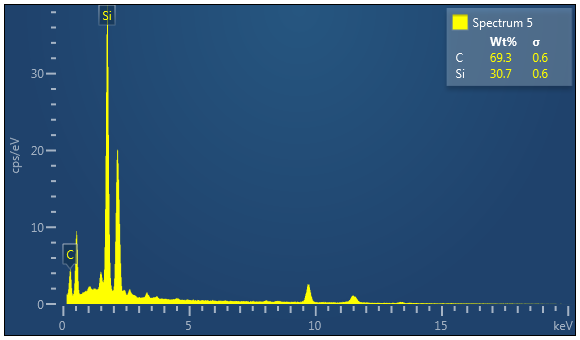

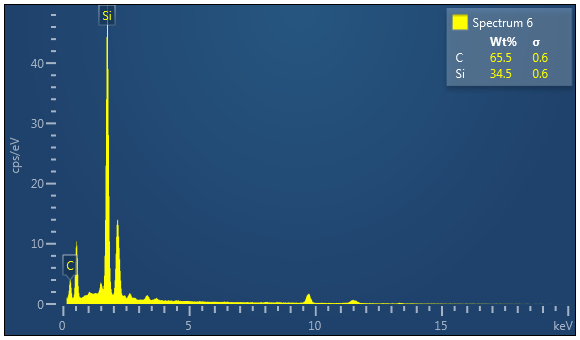


**Figure S9.** Elemental composition as determined by SEM-EDS mapping for CSC600.


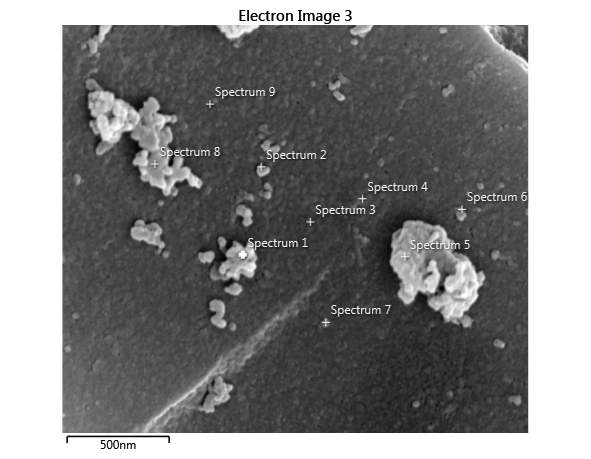


**Figure S10.** SEM-EDS mapping for CSC800.


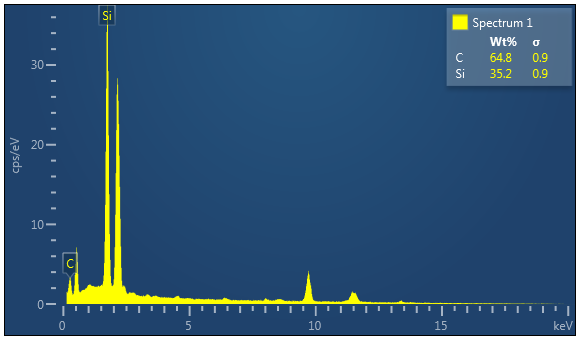

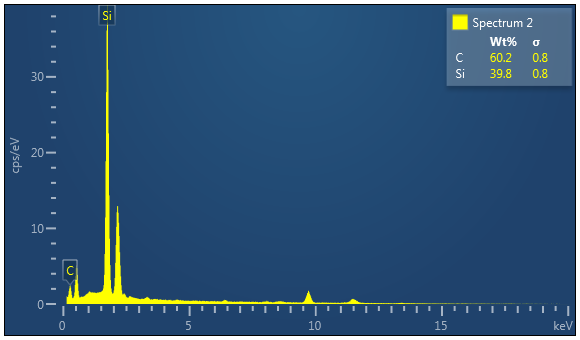

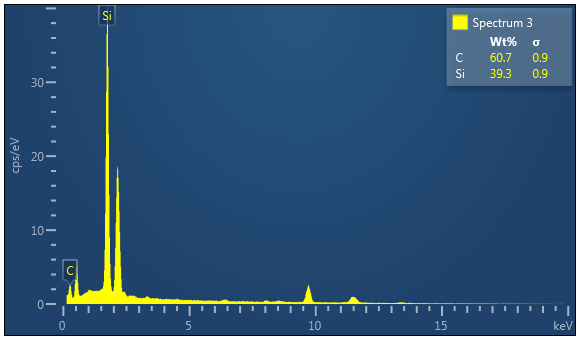

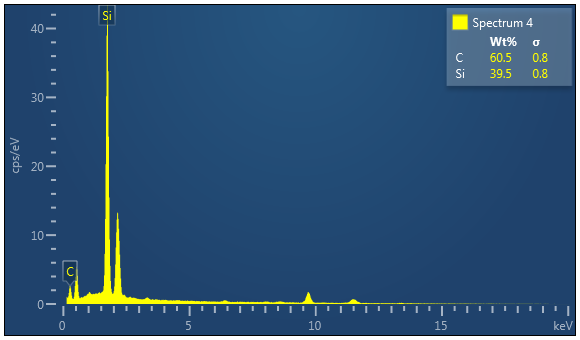

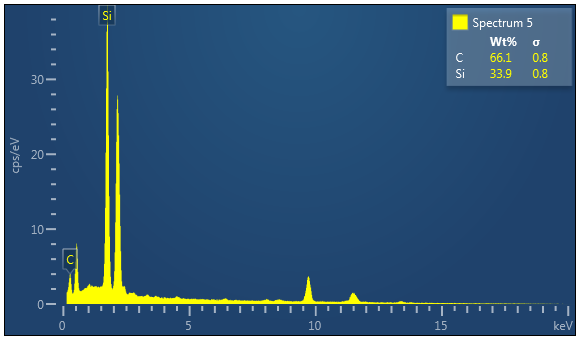

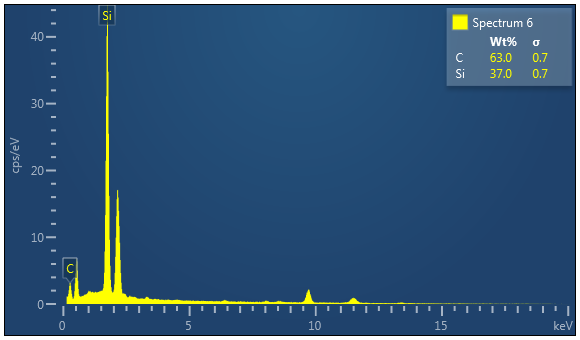

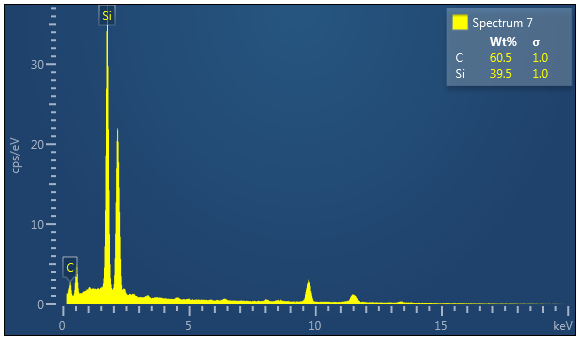


**Figure S11.** Elemental composition as determined by SEM-EDS mapping for CSC800.


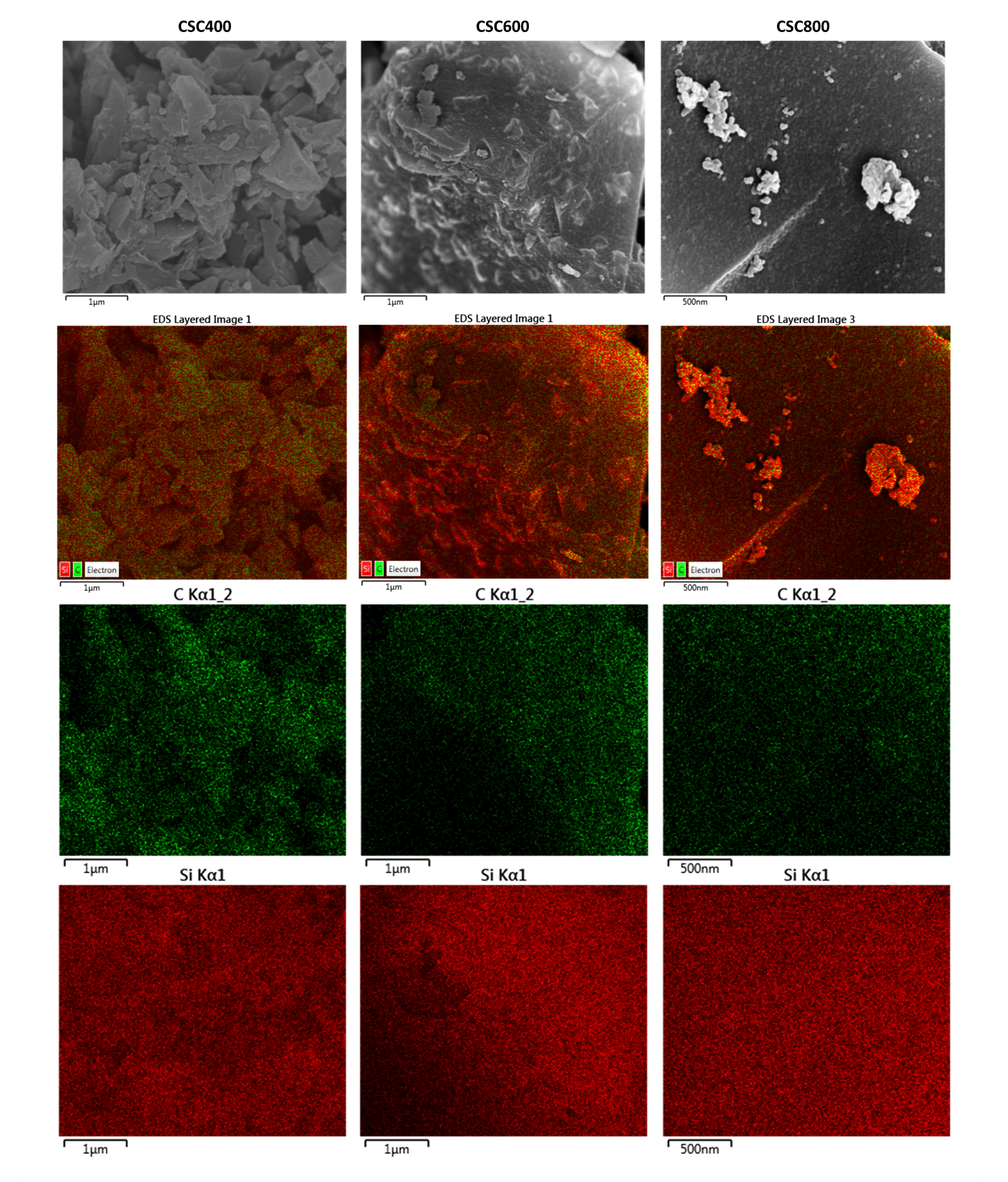


**Figure S12.** SEM-EDS mapping for CSC400, CSC600 and CSC800.


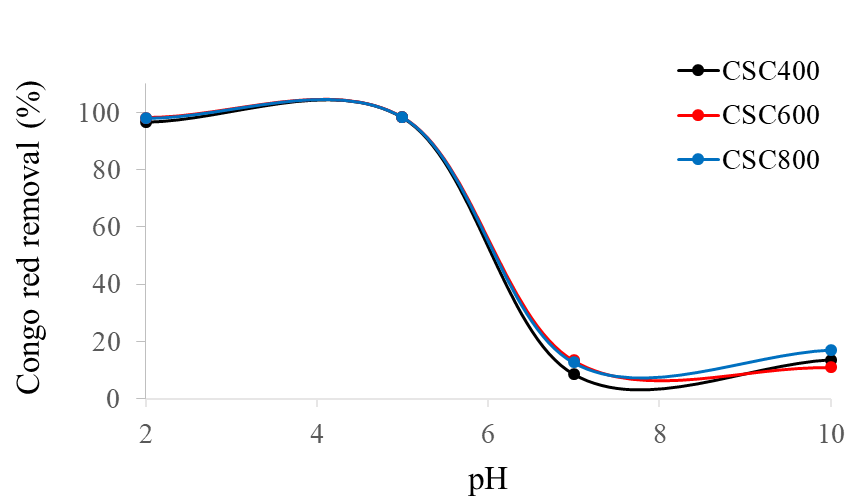


**Figure S13.** Effect of initial pH on the adsorption of congo red on CSCs.
